# Industry-sponsored clinical research outside high-income countries: an empirical analysis of registered clinical trials from 2006 to 2013

**DOI:** 10.1186/s12961-015-0019-6

**Published:** 2015-06-05

**Authors:** Srinivas Murthy, Kenneth D. Mandl, Florence T. Bourgeois

**Affiliations:** Department of Pediatrics, University of British Columbia, 4500 Oak Street, V6H 3V4 Vancouver, BC Canada; Division of Critical Care, BC Children’s Hospital, Vancouver, BC Canada; Center for Biomedical Informatics, Harvard Medical School, Boston, MA USA; Children’s Hospital Informatics Program, Boston Children’s Hospital, 320 Longwood Avenue, 02115-5737 Boston, MA USA; Division of Emergency Medicine, Boston Children’s Hospital, Boston, MA USA; Department of Pediatrics, Harvard Medical School, Boston, MA USA

**Keywords:** Clinical trials, Global health, Pharmaceutical companies

## Abstract

**Background:**

Industry-sponsored clinical trials, in the past performed almost exclusively in more developed countries, now often recruit participants globally. However, recruitment from outside high-income countries may not represent the ultimate target population for the intervention. Clinical trial registries provide an opportunity to quantify and examine the type of clinical research performed in various geographic regions. We sought to characterize industry-sponsored randomized controlled trials conducted in high-income countries and to compare these trials to those performed outside high-income countries.

**Methods:**

Clinical trial data on all industry-funded randomized controlled trials conducted between 2006 and 2014 were obtained from the registry ClinicalTrials.gov. Trials were classified according to their study sites as conducted in high or non-high income countries, and data on trial characteristics were collected.

**Results:**

Of 22,511 relevant trials, a total of 6,085 (27.0 %) trials included study sites outside a high-income country, and 2,045 (9.1 %) were conducted exclusively outside high-income countries. Of country groups, Central Europe had the greatest number of trials (3,127), followed by Eastern Europe (2,075). The percentage of trials with study sites outside high-income countries remained relatively constant over the study period. Studies with sites outside high-income countries tended to recruit more participants (median enrolled participants 265 vs. 71, *P* <0.001), to be longer (median study duration 20 vs. 13 months, *P* <0.05), and to study more advanced phase interventions (Phase 3 or 4 trial 58 % vs. 33 %, *P* <0.001).

**Conclusions:**

More than a quarter of industry-sponsored trials include participants from outside high-income countries and this rate remained stable over the 7-year study period. Trials conducted outside high-income countries tend to be larger, have a longer duration, and study later phase interventions compared to studies performed exclusively in high-income countries.

**Electronic supplementary material:**

The online version of this article (doi:10.1186/s12961-015-0019-6) contains supplementary material, which is available to authorized users.

## Background

Industry-sponsored clinical research has traditionally been performed in high-income countries, given the established research infrastructure and the geographic location of major pharmaceutical companies. Over recent decades, however, globalization has led to the extension of industry-sponsored clinical research outside higher income regions [1–3], with approximately one-third of large company-sponsored phase III trials being conducted exclusively outside the United States of America. Indeed, the total number of countries contributing results in major clinical trial publications between 1995 and 2005 has doubled [4, 5].

A number of factors contribute to the trend towards more international study sites. First, the global burden of disease is predominantly centred outside higher income regions, potentially accelerating trial recruitment for the large sample sizes required [6–8]. Secondly, increasingly complex regulatory environments in higher-income regions may slow down trial initiation and performance [9]. Thirdly, trials conducted in certain non-higher income countries, such as Russia, Argentina or China, may cost half the price of trials performed in the United States or Western Europe [10]. Finally, recognition of the growing market share of less-developed regions may provide added incentive to have drugs tested and approved in these countries [11].

This shift is not without controversies. Due to varying regulatory and legal environments, clinical trials performed outside high-income countries are presumed to be different in design and conduct [12, 13]. Clinical trial results from outside high-income countries may not be applicable to high-income countries due to differences in treatment effect sizes, rates of publication biases, and genetically different populations [1, 3, 12–18]. Additionally, ethical concerns about trial conduct outside higher income regions persist, including access to study interventions after trials are concluded and research misconduct [2, 5, 18].

Our aim was to describe current patterns regarding the globalization of industry-sponsored clinical trials, including the number of trials performed in different geographic regions and the collaboration between these regions. Our secondary aim was to characterize and compare trials performed in high- and non-high-income countries.

## Methods

### Study design

We performed a cross-sectional analysis of industry-sponsored clinical trials registered in ClinicalTrials.gov. This registry is a USA-based registry of clinical trials that represents as many as 86 % of all trial registrations [19]. It has been used to assess various aspects of clinical research activity, including correlation with disease burden, quality of clinical trials, and publication bias [20–22].

We identified all interventional trials with any funding by industry with a start date between January 1, 2006 and February 19, 2014 (date of data download). Trials studying a drug, device, biologic or dietary supplement, and employing a randomized design were selected. Trials without information on study location were excluded. Ethical approval was not obtained, given the nature of the study.

### Data extraction

We extracted data for each trial on study start and completion dates, allocation strategy, masking, trial phase, estimated enrolment number, major condition group, participant age eligibility, funding source, study site locations and number of sites, completion status, and posting of trial results. Information was collected on both lead and secondary funding sources. Condition groups were defined by the investigators for easily-defined conditions with a large disease burden (Additional file 1).

### Country classification

**Fig. 1:**
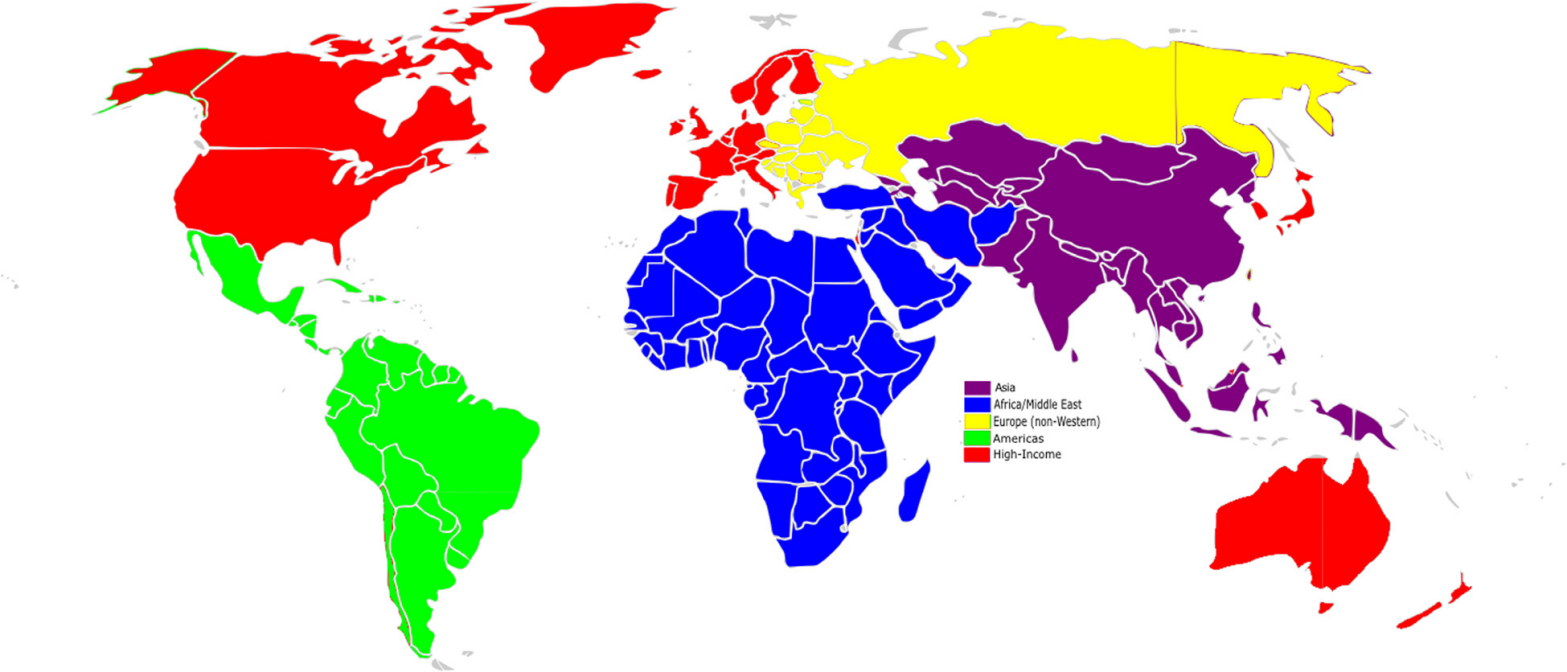
Global map of regions and major geographic groups

Countries were geographically classified according to the categorization used in the Global Burden of Disease project which classifies countries into 21 regions and these regions into five major geographic groups (Fig. 1), available at http://ghdx.healthdata.org/country_profiles [23]. The major groups are Asia, Africa/Middle East, Europe (non-Western), Americas, and High-income regions. The regions classified as High-income are High-income Asia Pacific, High-income North America, Australasia, and Western Europe. We considered all countries falling into one of these regions as high-income and all others as non-high income.

This categorization allows for both geographic and economic stratification, and is consistent with both World Bank income-based definitions and established clinical research infrastructure [13]. Countries were classified based on all the study sites listed in the registry record. Trials with study sites in both income regions were included in both.

### Data analysis

Descriptive analyses were performed quantifying study sites and trials and describing trial characteristics by region and major geographic group. Differences were compared with *χ*^2^ and Mann–Whitney tests. Time series analysis was performed with regression analysis. All statistical analyses were performed with R (R: A Language and Environment for Statistical Computing, version 3.1.1, Vienna Austria, 2014).

## Results

**Fig. 2:**
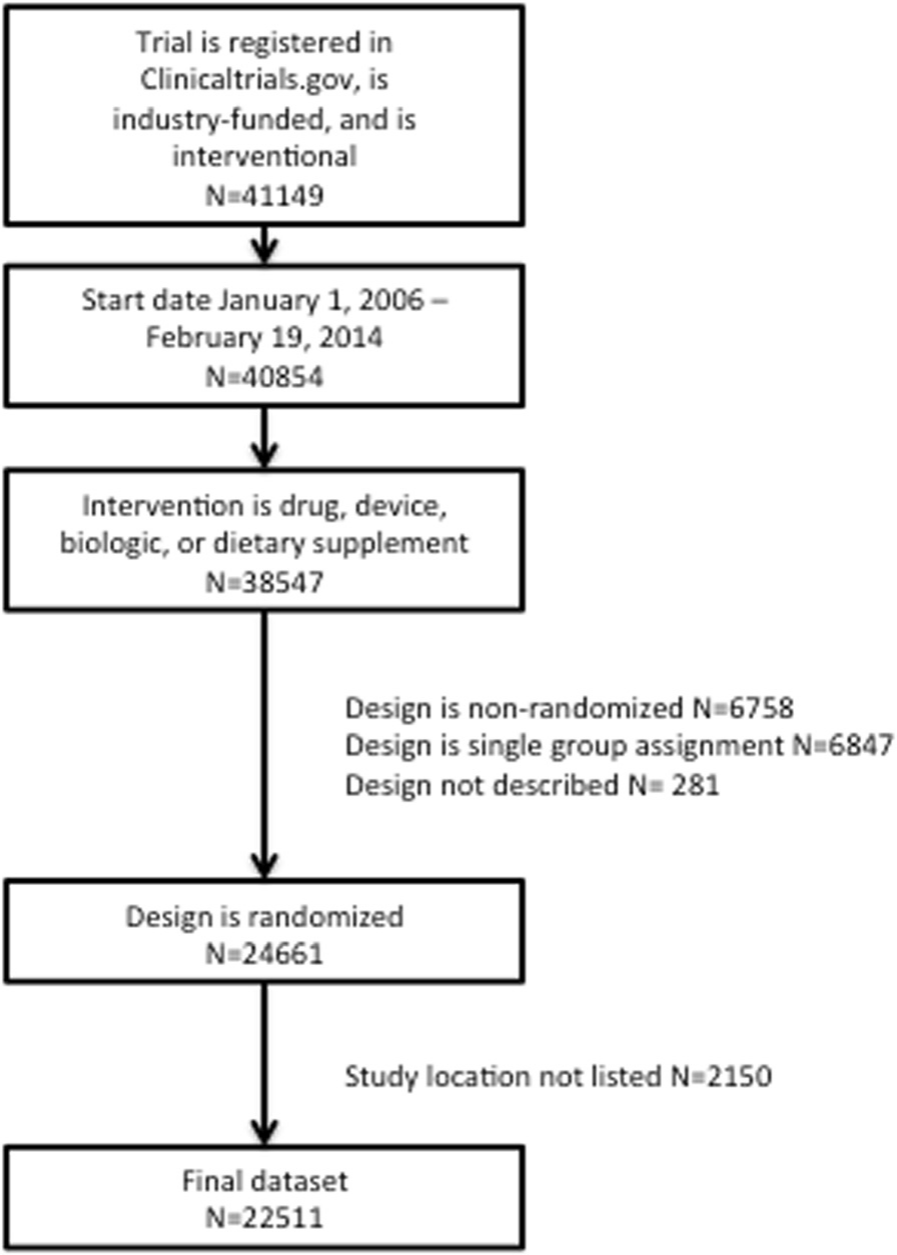
Flowchart of included trials

There were 41,149 industry-funded, interventional trials registered in ClinicalTrials.gov with start dates during the study period. Of these, 22,511 were randomized and information on study site location was included (Fig. 2).

A total of 2,045 (9.1 %) trials were conducted exclusively in non-high-income countries and an additional 4,040 (17.9 %) included sites in both high- and non-high-income countries (Table 1). The remainder of the trials (73.0 %) were conducted exclusively in high-income countries. Central Europe represented the less-developed country region with the greatest number of trials (N = 3127), followed by Eastern Europe (N = 2075) and East Asia (N = 1742). When adjusted for population, the country regions Central Europe, Southern Latin America, and Southern Sub-Saharan Africa represented the non-high-income regions with the greatest number of trials (Table 1).

**Table 1:** Clinical trial sites among industry-sponsored trials registered in ClinicalTrials.gov

Major geographic groups and country regions	Total number of trials ^a^ (N = 22,511)	Trials conducted exclusively outside high-income regions ^a^, N (%) (N = 2,045)	Total number of study sites	Trials/10 million population ^a^
**America**	2,245	445 (19.8)	22,296	36.7
Andean Latin America	444	22 (5.0)	1,507	78.2
Caribbean	138	28 (20.3)	614	35.4
Central Latin America	1,321	143 (10.8)	6,902	53.7
Southern Latin America	1,139	69 (6.1)	7,239	182.3
Tropical Latin America	1,067	231 (21.6)	6,034	51.5
**Middle East/Africa**	1,447	203 (14.1)	8,294	10.1
Eastern Sub-Saharan Africa	55	43 (78.2)	197	1.4
North Africa and Middle East	727	85 (11.7)	2,875	14.6
Central sub-Saharan Africa	57	11 (19.3)	41	5.7
Southern sub-Saharan Africa	911	60 (6.4)	4,903	121.8
Western sub-Saharan Africa	97	39 (40.2)	278	2.6
**Asia**	2,845	1198 (42.1)	23,448	7.6
Central Asia	107	19 (17.8)	291	12.4
East Asia	1,742	728 (41.8)	11,502	12.6
Oceania	39	2 (5.1)	16	40.6
South Asia	1,157	324 (20.0)	7,234	7.1
Southeast Asia	867	161 (18.6)	4,405	13.7
**Europe (non-Western)**	3,536	308 (8.7)	58,960	109.0
Eastern Europe	2,075	159 (7.7)	20,685	99.6
Central Europe	3,127	200 (6.4)	38,275	269.0
**More-developed**	20,466	–	378,331	207.5
Australasia	1,775	–	9,718	643.1
Western Europe	8,591	–	123,019	202.5
High-income Asia Pacific	2,859	–	23,441	155.9
High-income North America	12,992	–	222,153	369.8

^a^ Trials with study sites in more than one country region were included separately in each region

^b^ Population data from World Bank, 2013. Available at data.worldbank.org

In terms of number of study sites, 23 % of all sites were outside high-income countries. Trials conducted in Asia were the most likely to recruit exclusively outside high-income countries (42.1 % of all trials with sites in Asia) followed by the Americas (19.8 %).

**Fig. 3:**
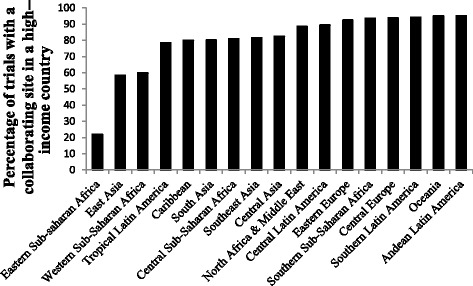
Percentage of trials performed in non-high income regions with a collaborating site in a high-income country

Among the major geographic groups, Europe (non-Western) had the highest rate of collaboration with the high-income regions, with 91.3 % of trials conducted having a high-income collaborating site, while trials with sites in Asia had the lowest rate, with 57.9 % of trials including a site in a high-income region. Among the country regions, trials in Eastern sub-Saharan Africa, East Asia, and Western sub-Saharan Africa were most frequently conducted without collaborating sites in high-income countries, while trials with study sites in Southern Latin America, Oceania, and Andean Latin America had the highest rates of collaboration with high-income countries (Fig. 3).

**Fig. 4:**
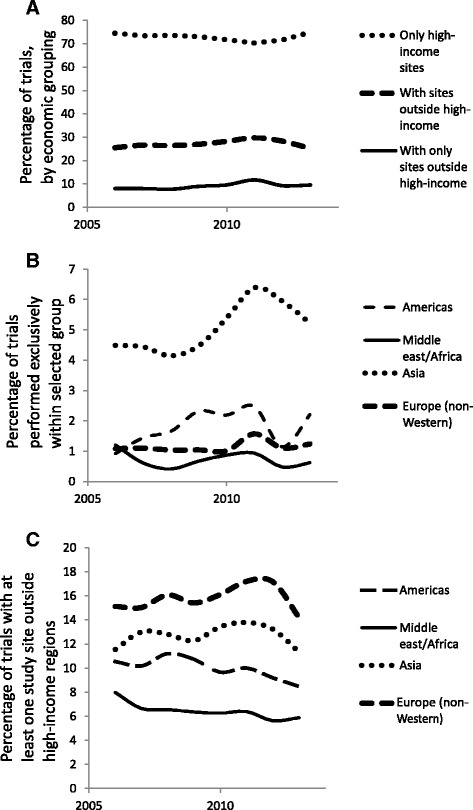
Temporal trends in trial performance by geographic group. **a** Percentage of trials performed in high and non-high-income geographic groups from 2006 to 2013; (**b**) Percentage of trials performed exclusively outside high-income major geographic group; (**c**) Percentage of trials performed with at least one study site outside the high-income geographic group. There were significant decreases in trials performed with sites in Middle East/Africa or the Americas (*P* = 0.01 and 0.02, respectively)

Figure 4 shows trends in the rate of trials with study sites in the different geographic groups. The percentage of trials with study sites in non-high-income regions remained relatively constant over the study period (Fig. 4a). Similarly, there were no trends in the percentage of trials performed exclusively within non-high-income major geographic groups, although there was some fluctuation in rates, especially for the Americas and Asia (Fig. 4b). At the same time, decreases were observed in the percentage of trials with any study site in the Americas (*P* = 0.02) and in the Middle East/Africa (*P* = 0.01; Fig. 4c). Europe (non-Western) remained the non-high-income geographic group with the highest participation in trials, followed by Asia and the Americas.

Trials performed in high- and non-high-income countries (Table 2) differed. For trials performed exclusively within one of the five major geographic groups, those performed outside high-income regions enrolled significantly more participants than trials in the high-income group. Trials performed exclusively in the Americas, Middle East/Africa, or Asia were less likely to use double-blinding compared to those performed in high-income regions. A greater proportion of trials in the Americas, Asia, and Europe (non-Western) had industry as a lead sponsor, and all four non-high-income geographic groups were more likely to study late phase interventions than the high-income group.

**Table 2:** Characteristics of industry-sponsored clinical trials performed exclusively within one major geographic group ^a^

Study characteristic	Major geographic group				
	Americas N = 406	Middle East/Africa N = 163	Asia N = 1138	Europe (non-Western) N = 256	High-income regions N = 16,246
Median study sites, N (IQR)	1 ^c^	1 ^c^	1 ^c^	2 ^c^	1
	(1–2)	(1–3)	(1–4)	(1–9)	(1–9)
Median subjects enrolled, N (IQR)	100 ^c^	183 ^c^	142 ^c^	120 ^c^	71
	(48–200)	(90–400)	(50–300)	(47–259)	(33–188)
Double-blinded, N (%)	191 ^c^	73 ^c^	502 ^c^	168	9938
	(46.9)	(44.7)	(44.1)	(65.6)	(61.1)
Industry as lead funder, N (%)	345 ^c^	115 ^c^	998 ^c^	236 ^c^	13 133
	(84.8)	(70.5)	(87.7)	(92.2)	(80.8)
Phase 3 or 4, N (%)	250 ^c^	73 ^c^	589 ^c^	104 ^c^	5345
	(61.6)	(44.7)	(51.7)	(40.6)	(32.9)
Paediatric ^b^ N (%)	42 ^c^	51 ^c^	120 ^c^	28 ^c^	699
	(10.3)	(31.3)	(10.5)	(10.9)	(4.2)

^a^ 18,209 (80.9 %) of all trials were conducted exclusively in one region and are included in the table

^b^ Defined as listed maximum age <19, or if listed maximum age above 19, median age <19. If no maximum age listed, trial assumed to be adult

^c^ *P* <0.05 by *χ* ^2^ or Mann–Whitney testing when compared with high-income regions

Additionally, trials performed with at least one study site in a non-high-income country had a larger median number of sites (25 vs. 1, *P* <0.001), enrolled a greater median number of participants (265 vs. 71, *P* <0.001), and were more likely to have industry as the lead funder (94.6 % vs. 80.8 %, *P* <0.001) compared to trials with sites exclusively in high-income countries (Table 3). Trials with sites in non-high-income countries were also more likely to be phase 3 or 4 trials (58.0 % vs. 32.9 %, *P* <0.001) and have a longer median duration (20.3 months vs. 13.2 months, *P* <0.05) than trials performed in more-developed countries. The proportion of trials with sites outside high-income regions was highest for trials studying diabetes (54.4 % of trials), with healthy volunteer studies having just 9.9 % of trials conducted with a site outside a high-income region (Table S1, Additional file 1). Of the 1,236 trials performed in children, 537 (43.4 %) had a site outside a high-income country with similar trends existing regarding rate of double-blinding, advanced phase, and study size when compared with all other trials (Additional file 1: Table S2).

**Table 3:** Characteristics of industry-sponsored clinical trials by study site income level

Trial characteristic	All trials N = 22,511	Trials with study sites outside high-income countries, N = 6,085	Trials with study sites exclusively in high-income countries, N = 16,426	*P* value ^c^
Median study sites, N (IQR)	3 (1–20)	25 (4–71)	1 (1–9)	<0.001
Median participants enrolled, N (IQR)	100 (40–283)	265 (104–554)	71 (33–186)	<0.001
Double-blinded, N (%)	13,883 (61.7)	3,945 (64.8)	9,938 (61.1)	<0.001
Industry as lead funder, N (%)	18,891 (83.9)	5,758 (94.6)	13,133 (80.8)	<0.001
Sponsored by one of the 10 largest pharmaceutical companies ^a^ N (%)	5,522 (24.5)	2,182 (35.9)	3,340 (20.3)	<0.001
Phase 3 or 4, N (%)	8,877 (39.4)	3,532 (58.0)	5,345 (32.9)	<0.001
Paediatric ^b^ N (%)	1,236 (5.5)	537 (8.8)	699 (4.2)	<0.001
Median trial length, months ^d^	15.2 (7.1–27.4)	20.3 (12.2–34.5)	13.2 (6.0–25.3)	<0.05
Completed, N (%) ^e^	11,703/16,296 (71.8)	3,011/4,392 (68.5)	8,692/11,904 (73.0)	<0.001
With results posted on ClinicalTrials.gov, N (%) ^f^	3,142/11,703 (26.8)	1,001/3,011 (33.2)	2,141/8,692 (24.6)	<0.001

^a^ Pfizer, Novartis, Sanofi, Merck, Roche, GlaxoSmithKline, Abbott, AstraZeneca, Amgen, Eli Lilly as defined by total revenues at Forbes.com

^b^ Defined as listed maximum age <19, or if listed maximum age above 19, median age < 19. If no maximum age listed, trial assumed to be adult. See Additional file 1 for further details (Table S2)

^c^ *χ* ^2^ or Mann–Whitney for medians

^d^ Available in 21 223 trials

^e^ Among trials started before January 1, 2012

^f^ Among trials labelled as complete and started before January 1, 2012

## Discussion

We found that more than a quarter of trials registered in ClinicalTrials.gov are recruiting participants in non-high-income countries, with the majority of these trials enrolling in both high- and non-high-income countries. Of the non-high-income geographic groups, Europe (non-Western) and Asia have the greatest proportion of trials.

Trials performed in different regions of the world differ substantially; those conducted in non-high-income countries recruit from a greater number of study sites and study more advanced-phase interventions. Trials with sites in non-high-income countries also tend to recruit more subjects when compared to those conducted exclusively in high-income countries, indicating that subjects from non-high-income countries may contribute substantially to trial results. This is consistent with the limited prior work examining potential differences in trial characteristics conducted in high- and non-high-income countries [24–26].

There are a number of factors underlying the ongoing trend of recruiting participants from non-high-income countries. The lower cost is likely a key driver, as evidenced by the high number of sites and large trial sizes among trials conducted in non-high-income regions such as Europe (non-Western) and Asia [10]. In addition, the ease of recruitment of treatment-naïve patients with chronic disease makes non-high-income countries appealing for subject recruitment [27].

Anthropological analyses of international research have described three phases, where the first is a massive influx of research infrastructure into a non-high-income region, the second an increase in the regulatory environment with more stringent oversight, and the third, a shift towards specific demands about the nature of the proposed research by local researchers and patients [28]. It is uncertain where on this continuum research activity currently lies among the regions examined, but the relatively stable activity in the regions may indicate that they may have entered the second or third phases with greater local involvement. Prior studies documenting substantial increases in trial activity in many of these non-high-income regions up through 2005 would further support this possibility [4, 5].

A number of factors should be considered when extrapolating trial results from non-high-income clinical settings to high-income countries. For one, ethical standards differ by region, despite the existence of international frameworks, leading to potential exploitation of participants [1, 29]. Streamlining regulatory oversight across regions would allow for the maintenance of trial standards, negating the search for more lax regions in which to perform studies. In addition, prior work suggests that non-high-income countries have larger effect sizes for interventional studies, raising concerns that the populations in high-income countries may not experience the benefits predicted by premarket trials [13]. One postulated reason for this effect size difference is the narrow targeting of treatment-naïve patients with advanced phase interventions in non-high-income countries.

One of the limitations of this study is that there is no definitive classification for high- and non-high-income countries and, while we used a widely accepted categorization scheme, some of the countries may have been classified differently using other approaches. In addition, the accuracy of the data provided in ClinicalTrials.gov relies on investigators and we were not able to verify the information. There is also missing data in the registry, although we encountered only a small proportion of missing information for the variables of interest. Finally, although ClincialTrials.gov is the largest and most comprehensive trial registry, it is possible that some trials were not registered or were registered in other, country-specific registries. However, this likely represents a very small number of trials since all pharmaceutical companies seeking Food and Drug Administration approval of a drug (which is required for marketing in the USA) must register their trials in the ClinicalTrials.gov registry.

## Conclusions

More than a quarter of all pharmaceutical company trials recruit participants from non-high-income nations. The percentage of trials in non-high-income countries remained stable over the seven-year study period. There are a number of differences in the design and conduct of trials in high and non-high-income countries, including trials in non-high-income countries enrolling more participants and studying later phase interventions. The scientific implications of participant recruitment from these diverse geographic regions and of the differences in trial characteristics in different regions warrant further exploration.

## Additional file

Additional file 1: Table S1. Subgroup analyses of major conditions. Cardiovascular is defined as studies meeting search criteria for conditions involving coronary artery disease, cardiac dysfunction, or known risk factors for the above such as hypercholesterolemia and hypertension. Psychiatric trials were defined as studies meeting search criteria for common psychiatric disorders. Diabetes, asthma, and COPD were searched using their respective terms. **Table S2.** Trials involving children, defined as listed maximum age <19, or if listed maximum age above 19, median age <19. If no maximum age listed, trial assumed to involve adults. **Figure S1.** Top 10 pharmaceutical companies and studies with sites outside high-income regions, as defined by total revenues at Forbes.com.

## References

[1] Lang T, Siribaddana S (2012). Clinical trials have gone global: is this a good thing?. PLoS Med.

[2] MacMahon S, Perkovic V, Patel A (2013). Industry-sponsored clinical trials in emerging markets: time to review the terms of engagement. JAMA.

[3] Lorenzo C, Garrafa V, Solbakk JH, Vidal S (2010). Hidden risks associated with clinical trials in developing countries. J Med Ethics.

[4] Khin NA, Yang P, Hung HM, Maung UK, Chen YF, Meeker-O’Connell A (2013). Regulatory and scientific issues regarding use of foreign data in support of new drug applications in the United States: an FDA perspective. Clin Pharmacol Ther.

[5] Glickman SW, McHutchison JG, Peterson ED, Cairns CB, Harrington RA, Califf RM (2009). Ethical and scientific implications of the globalization of clinical research. N Engl J Med.

[6] Lozano R, Naghavi M, Foreman K, Lim S, Shibuya K, Aboyans V (2012). Global and regional mortality from 235 causes of death for 20 age groups in 1990 and 2010: a systematic analysis for the Global Burden of Disease Study 2010. Lancet.

[7] Pereira TV, Horwitz RI, Ioannidis JP (2012). Empirical evaluation of very large treatment effects of medical interventions. JAMA.

[8] Siontis GC, Ioannidis JP (2011). Risk factors and interventions with statistically significant tiny effects. Int J Epidemiol.

[9] Yusuf S (2004). Randomized clinical trials: slow death by a thousand unnecessary policies?. CMAJ.

[10] Kaitin KI (2010). The landscape for pharmaceutical innovation: drivers of cost-effective clinical research. Pharm Outsourcing.

[11] Imran M, Najmi AK, Rashid MF, Tabrez S, Shah MA (2013). Clinical research regulation in India-history, development, initiatives, challenges and controversies: Still long way to go. J Pharm Bioallied Sci.

[12] Smith R (2002). Publishing research from developing countries. Stat Med.

[13] Panagiotou OA, Contopoulos-Ioannidis DG, Ioannidis JP (2013). Comparative effect sizes in randomised trials from less developed and more developed countries: meta-epidemiological assessment. BMJ.

[14] Zhang D, Freemantle N, Cheng KK (2011). Are randomized trials conducted in China or India biased? A comparative empirical analysis. J Clin Epidemiol.

[15] Vickers A, Goyal N, Harland R, Rees R (1998). Do certain countries produce only positive results? A systematic review of controlled trials. Control Clin Trials.

[16] O’Connor CM, Fiuzat M, Swedberg K, Caron M, Koch B, Carson PE (2011). Influence of global region on outcomes in heart failure beta-blocker trials. J Am Coll Cardiol.

[17] Goldstein DB, Tate SK, Sisodiya SM (2003). Pharmacogenetics goes genomic. Nat Rev Genet.

[18] Ana J, Koehlmoos T, Smith R, Yan LL (2013). Research misconduct in low- and middle-income countries. PLoS Med.

[19] Viergever RF, Ghersi D (2011). The quality of registration of clinical trials. PLoS One.

[20] Zarin DA, Tse T, Williams RJ, Califf RM, Ide NC (2011). The ClinicalTrials.gov results database–update and key issues. N Engl J Med.

[21] Bernardez-Pereira S, Lopes RD, Carrion MJ, Santucci EV, Soares RM, de Oliveira AM (2014). Prevalence, characteristics, and predictors of early termination of cardiovascular clinical trials due to low recruitment: insights from the ClinicalTrials.gov registry. Am Heart J.

[22] Bourgeois FT, Olson KL, Ioannidis JP, Mandl KD (2014). Association between pediatric clinical trials and global burden of disease. Pediatrics.

[23] Global Health Data Exchange: Country Profiles http://ghdx.healthdata.org/country_profiles

[24] Fiuzat M, Califf RM (2011). Conduct of clinical trials in acute heart failure: regional differences in heart failure clinical trials. Heart Fail Clin.

[25] Shirotani M, Kurokawa T, Chiba K (2014). Comparison of global versus Asian clinical trial strategies supportive of registration of drugs in Japan. J Clin Pharmacol.

[26] Nair SC, Ibrahim H, Celentano DD (2013). Clinical trials in the Middle East and North Africa (MENA) Region: grandstanding or grandeur?. Contemp Clin Trials.

[27] Emerging Markets Clinical Trials: Eastern Europe (PH145); Cutting Edge Information. North Carolina; 2010. https://www.cuttingedgeinfo.com/research/clinical-development/central-eastern-europe-trials/. Accessed June 2, 2015.

[28] Petryna A (2009). When experiments travel: clinical trials and the global search for human subjects: Princeton university press.

[29] ICH – Harmonization for Better Health. International Conference on Harmonisation of Technical Requirements for Registration of Pharmaceuticals for Human Use. Efficacy guidelines. http://www.ich.org/products/guidelines/efficacy/article/efficacy-guidelines.html. Accessed June 2, 2015.

