# Supplementary material for: Industry-sponsored clinical research outside high-income countries: an empirical analysis of registered clinical trials from 2006 to 2013
Source: Health Res Policy Syst. 2015 Jun 5;13:28. doi: 10.1186/s12961-015-0019-6 (PMC4465475; doi:10.1186/s12961-015-0019-6)
Supplement: Additional file 1: Table S1. — Subgroup analyses of major conditions. Cardiovascular is defined as studies meeting search criteria for conditions involving coronary artery disease, cardiac dysfunction, or known risk factors for the above such as hypercholesterolemia and hypertension. Psychiatric trials were defined as studies meeting search criteria for common psychiatric disorders. Diabetes, asthma, and COPD were searched using their respective terms. Table S2. Trials involving children, defined as listed maximum age <19, or if listed maximum age above 19, median age <19. If no maximum age listed, trial assumed to involve adults. Figure S1. Top 10 pharmaceutical companies and studies with sites outside high-income regions, as defined by total revenues at Forbes.com. [file 12961_2015_19_MOESM1_ESM.docx]

|  | Total | With site outside high-income countries  (%) |
| --- | --- | --- |
| Cardiovascular | 1468 | 428 (29.1) |
| Diabetes | 1652 | 900 (54.4) |
| Asthma/COPD | 925 | 314 (33.9) |
| Psychiatric | 807 | 258 (31.9) |
| Healthy volunteers | 1861 | 184 (9.9) |

Table S1: Subgroup analyses of major conditions.

Cardiovascular is defined as studies meeting search criteria for conditions involving coronary artery disease, cardiac dysfunction, or known risk factors for the above such as hypercholesterolemia and hypertension. Psychiatric trials were defined as studies meeting search criteria for common psychiatric disorders. Diabetes, asthma and COPD were searched using their respective terms.

| PEDIATRIC STUDIES | All trials  N=1236 | Trials with study sites in high-income countries only N=699 | Trials with study sites outside high-income countries  N= 537 | P-value |
| --- | --- | --- | --- | --- |
| Median study sites, N (IQR) | 5 (1-22) | 3 (1-16) | 7 (2-34) | P<0.05 |
| Median subjects enrolled, N (IQR) | 178 (60-430) | 102 (44-300) | 300 (120-660) | P<0.001 |
| Double-Blinded (%) | 772 (62.4) | 454 (64.9) | 318 (59.2) | P<0.05 |
| Industry as lead funder (%) | 1023 (82.7) | 525 (75.1) | 498 (92.7) | P<0.001 |
| Phase 3,3/4,4 (%) | 711 (57.5) | 361 (51.6) | 350 (65.2) | P<0.001 |

Table S2: Trials involving children, defined as listed maximum age <19, or if listed maximum age above 19, median age <19. If no maximum age listed, trial assumed to be adult.

Figure S1: Top 10 Pharmaceutical companies and studies with sites outside high-income regions, as defined by total revenues at Forbes.com
